# Cost-effectiveness of oil and milk fortification by scale for reducing Vitamin A and Vitamin D deficiency in India

**DOI:** 10.1371/journal.pone.0331790

**Published:** 2025-09-08

**Authors:** Abhishek Kumar, Komal Ahluwalia, Anirudhan P. Edathil, Rajan Sankar, Nimmathota Arlappa, Sirimavo Nair, Mona Duggal, William Joe

**Affiliations:** 1 Department of Economics, School of Liberal Education, FLAME University, Lavale, Maharashtra, India; 2 Institute of Economic Growth, Delhi University Enclave (North Campus), Delhi, India; 3 Senior Advisor, Partnerships For Impact, Delhi, India; 4 Division of Public Health Nutrition, ICMR-National Institute of Nutrition (ICMR-NIN), Tarnaka, Hyderabad, Telangana, India; 5 Department of Foods and Nutrition, Faculty of Family and Community Sciences, M S University of Baroda, Vadodara, Gujarat, India; 6 Indian Council of Medical Research, National Institute for Research in Reproductive and Child Health, Mumbai, Maharashtra, India; Indian Institute of Information Technology, INDIA

## Abstract

**Background:**

Although broad-scale data might suggest low prevalence, millions of children in India still suffer from Vitamin A and Vitamin D deficiencies despite India’s existing guidelines for Vitamin A deficiency. To address the issue, the Government of India has recommended fortification of oil and milk to improve Vitamin A and Vitamin D consumption. However, there is limited information on the health benefits and cost-effectiveness of fortifying oil and milk at scale.

**Objective:**

To estimate the health benefits and cost-effectiveness of supplementation programme and fortification of milk and oil among children under 5 years, pregnant women, women in the reproductive age group, and the elderly.

**Data and methods:**

To measure the health benefits associated with supplementation and fortification of oil and milk, the number of DALYs that are currently lost due to Vitamin A and Vitamin D deficiencies were estimated. For Vitamin A related mortality, a reduction of 4%, 12% and 23% were assumed while the assumptions for estimating morbidity benefits were derived from Global Burden of Disease. For the costing exercise, we considered the following two scenarios: (1) high-dose vitamin A supplementation for children and pregnant women; (2) industrial fortification of oil for children, pregnant women, women in the reproductive age group, and the elderly.

**Results:**

Overall, intervention related to Vitamin A could avert 1,119,044 Years of Life Lost (YLLs) at a 23% reduction, 194,616 YLLs at 4%, and 583,849 YLLs at 12% and 28,534 YLDs. Intervention related to Vitamin D could avert 99,219 YLDs. The total cost for supplying supplements to approximately 109,965 thousand children and 26,920 thousand pregnant women is around 26 million USD. The cost to fortify is 7.6 million USD for oil and 9.8 million USD for milk fortification for children and women. The overall cost effectiveness ratio of the fortification programme is 150.

**Conclusion:**

Fortification could emerge as a potentially superior long-term solution, considering the widespread consumption of oil and milk, offering a broader reach to the population.

## 1. Introduction

Vitamin A (retinol) and Vitamin D (cholecalciferol) deficiency can lead to various health issues in both children and adults. In children, a lack of vitamin A may result in night blindness, increased vulnerability to infections such as measles, diarrhea, pneumonia and respiratory diseases, and delayed growth [1–3]. In pregnant women, vitamin A deficiency can manifest as night blindness and lead to preterm delivery and maternal anemia [4]. And, Vitamin D deficiency in children can lead to rickets, and increases the risk of osteomalacia and osteoporosis among adults [5].

As per the Comprehensive National Nutrition Survey (CNNS, 2016–18), the inflammation-adjusted prevalence of Vitamin A deficiency in school-age children and adolescents was 19.3% and 14.4%, respectively, and this was greater than 20 per cent in seven and four states for children and adolescents, respectively [6]. The CNNS secondary data revealed that VAD continues to be a serious public health concern in 17 states of India [7]. While the Vitamin D deficiency was 18.2% and 23.9% in school-age children and adolescents [8], the prevalence of Vitamin A and Vitamin D deficiencies was significantly higher among children with lower socio-economic status [8]. The proportion of rural, tribal, and urban children in India with inadequacy (≤70% of Recommended Dietary Intakes) of intakes of oils and milk ranges from a low of 86.2 to a high of 100%. The dietary inadequacy of vitamin A intake was also poor, ranging from 86–93 per cent [9]. While the prevalence of Vitamin A and Vitamin D deficiency appears to be low on a broad scale, it still impacts millions of children in India, exhibiting notable variations across different states. Furthermore, population estimates for Vitamin A and Vitamin D deficiencies are unavailable, limiting our understanding of the magnitude of the problem.

Although India has a national program for Vitamin A, there is no equivalent program for Vitamin D. The Vitamin A supplementation programme was rolled out in the 1970s to prevent childhood blindness in children, but the programme has suffered from poor outreach [10]. As per India’s policy framework, every infant 6–11 months and children 1–5 years are to be administered vitamin A every 6 months [10]. The recommended schedule is as follows: the first dose of 100,000 IU is administered with measles vaccination at 9 months and subsequently every six months, doses of 200,000 IU each. A child must receive a total of 9 oral doses of vitamin A by its fifth birthday [10]. According to the recently released National Family Health Survey (NFHS-5, 2019–21), only one-third of the children aged 12–23 months received vitamin A supplementation as compared with only one-fifth of the children aged 24–35 months, and half of the children have reported consuming vitamin A-rich foods and vegetables [11,12]. In fact, the consumption of Vitamin A is 50% among women in the 15–49 age group [11]. Although the coverage of Vitamin A supplementation has been increasing, the sub-optimal coverage and high deficiency indicated by various rounds of national surveys call for revisiting the design of the Vitamin A programme [6,8,10–13]. On the other hand, data collection for Vitamin D is not as comprehensive as Vitamin A. The issue of Vitamin D deficiency is less systematically addressed, although clinical guidelines have been issued by the Indian Academy of Pediatrics for infants and children [14].

Recently, in 2017, the Food Safety and Standards Authority of India (FSSAI, 2017) issued a recommendation advocating for the fortification of several staple food items, including wheat flour, rice, milk, edible oil, and salt [15]. The aim is to enhance the intake of essential micronutrients within the population. The history of oil fortification in India traces back to the 1950s when the government proposed fortifying Vanaspathi to enhance its nutritional value [13]. Presently, many prominent oil producers voluntarily fortify at least one of their brands to contribute to improved nutritional outcomes [15]. Additionally, the Ministry of Women and Child Development and the Ministry of Human Resource Development have recommended the incorporation of double-fortified salt (iron and iodine), wheat flour (fortified with iron, folic acid, and vitamin B-12), and edible oil (fortified with vitamin A and D) within their schemes, namely the Integrated Child Development Services (ICDS) and the Mid-day Meal (MDM) Scheme.

The Food Safety and Standards (Fortification of Foods) Regulations, 2018, initially set a compliance deadline of January 1, 2019. However, the COVID-19 pandemic led to a postponement of this implementation. These were then amended and came into effect on September 22, 2021; food business operators were required to comply with these regulations by April 1, 2022. The regulations specify the fortification requirements for vegetable oil and species-identified milk. For industrially produced fortified vegetable oil, the levels of Vitamin A and Vitamin D should be 6 µg RE – 9.9 µg RE and 0.11 µg– 0.16 µg per g of oil respectively. And, the source of nutrient could be Retinyl acetate or Retinyl palmitate for Vitamin A and Cholecalciferol or Ergocalciferol for Vitamin D. Similarly, in the fortified milk the level of Vitamin A should be 270–450 μg RE and level of Vitamin D should be 5–7.5 µg [13]. The specified levels aim to enhance the micronutrient content of these food items in compliance with the FSSAI regulations. It is worth noting here that food fortification has been approved by several countries [15]. However, a systematic review has also highlighted that fortified oil might not be adequate to reduce the deficiencies [16]. In case of India, a study on Vitamin A reported that supplementation might be cost effective as compared to fortification but fortification might be desirable given its wider reach [17].

With this context, the primary aim of the study is to evaluate the cost-effectiveness of two interventions: vitamin A supplementation and the fortification of oil and milk with vitamin A and Vitamin D. Our analysis focuses on estimating the potential benefits that could result from enhanced coverage of vitamin A and Vitamin D, specifically targeting children under 5 years of age, women in reproductive age group (15–49 years) which are extremely vulnerable groups. Furthermore, we estimated the benefits of Vitamin D fortification for the elderly (60 years and above), who are likely to suffer from osteoporosis and are more prone to falls. We prioritized the elderly in analysis because age-related changes such as reduced skin synthesis, lower dietary absorption, and declining kidney function make older adults particularly vulnerable.

## 2. Data and methods

### Conceptual framework

**Fig 1:**
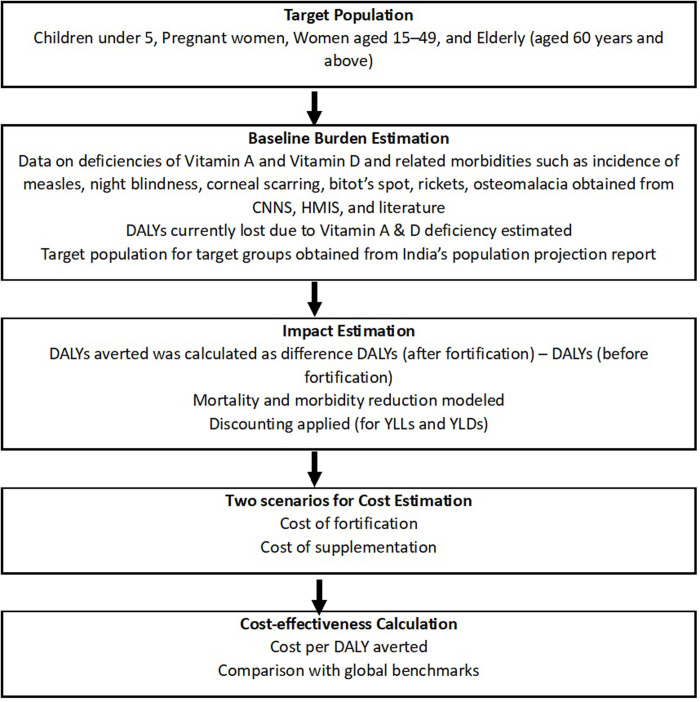
Analytical framework for estimating health and economic impact of Vitamin A and D fortification of milk and oil.

The analytical framework presented in Fig 1 outlines the step-by-step approach used to estimate the health and economic impact of vitamin A and D fortification in India. The first step was the identification of the target population groups—children under 5, pregnant women, women of reproductive age, and the elderly. These groups were selected because they are nutritionally vulnerable and because reliable data is more readily available for them through national surveys and administrative sources.

The next step involved estimation of the baseline burden of disease in terms of DALYs lost due to deficiencies, using nationally available data sources. We then modelled the potential impact of fortification in reducing this burden, followed by estimating associated costs for fortification and supplementation. Finally, the cost-effectiveness of the intervention using the cost per DALY averted, with scenario-based sensitivity analysis were conducted. We elaborate on the assumptions in the following section.

### Method – Benefits

To measure the health benefits associated with the fortification of oil and milk, the DALY framework was applied, which is widely used by the World Bank and the World Health Organization (WHO) to estimate the health benefits associated with various nutrition interventions [18–24]. This framework is used to estimate the mortality and disability-weighted morbidity associated with health conditions and is based on the following equation:


$$\text{DALYs}=\text{YLL}+\text{YLD}=\sum_{\text{j}}^{\text{\textbackslash{} }}{\text{T}}_{\text{j}}{\text{M}}_{\text{j}}\left(\frac{1-e^{-{rL}_{j}}}{r}\right)+\sum_{\text{j}}{\text{T}}_{\text{j}}{\text{I}}_{\text{ij}}D_{\text{ij}}(\frac{1-e^{-{rd}_{ij}}}{r})$$

Where:

YLD, years lived with disability;

YLL, years of life lost;

T_j_, total number of people in target group j/ Live births;

M_j_, the mortality rate associated with the deficiency in target group j;

r, discount rate for future life years;

L_j_, average remaining life expectancy for target group j;

I_ij_, incidence rate of functional outcome i in target group j;

D_ij_, disability weight for functional outcome i in target group j,

d_ij_, duration of functional outcome i in target group j.

Where functional outcomes refer to the observable effects or consequences of a particular health condition or intervention on an individual’s ability to perform activities.

To initiate the analysis, we first estimated the number of DALYs that are currently lost due to Vitamin A and Vitamin D deficiencies among children under 5 years, pregnant women, and women in the 15–49 age group. The number of deaths for children under five were calculated by multiplying the mortality rate obtained from NFHS with the number of live births. The number of years lived with disability was calculated as product of target population, disability weight and duration and incidence rate of the outcome for the population.

Next, we measured the number of DALYs that would still be lost when the fortification strategy is implemented. The net impact of fortification was measured by comparing the difference in DALYs estimated using the above two scenarios. The monetary value of DALY was estimated by multiplying the DALYs averted by per capita GDP (₹115746), taken as a proxy for the value of a statistical life.The data for per capita GDP was obtained from RBI database. DALYs averted were estimated as follows:


$${\text{YLL}}_{Averted}=\sum_{\text{j}}^{\text{\textbackslash{} }}{\text{T}}_{\text{j}}M_{j}^{Averted}(\frac{1-e^{{-rL}_{j}}}{r})$$

$${\text{YLD}}_{Averted}=\;\sum_{\text{j}}{\text{T}}_{\text{j}}I_{ij}^{Averted}D_{\text{ij}}(\frac{1-e^{{-rd}_{ij}}}{r})$$

Where $M_{j}^{Averted}$ and $I_{ij}^{Averted}$ refer to the reductions in mortality and morbidity rates.

Table 1 summarizes the parameter values used in the DALY formula. Vitamin A deficiency is associated with the following functional outcomes among children under five: measles and corneal scarring, with an attribution level of 10%. While 100% of night blindness and bitot’s spot is attributed to VAD. Vitamin A related functional outcomes also include night blindness, especially in pregnant women [25,26] or even permanent outcomes, such as corneal scarring [27,28]. Vitamin D deficiency is associated with rickets among children. The data on Vitamin D deficiency in children was obtained from CNNS while the data on Vitamin D deficiency among women was based on review of literature [8,29]. The data on measles was obtained from Health Management Information System database for the year 2022–2023. Prevalence was assumed to be 0.1% for night blindness and bitot’s spot. Incidence was calculated as prevalence divided by duration. For Corneal Scarring, incidence was calculated as the product of prevalence and live births divided by total population under five.

**Table 1:** Parameter values used in DALY formula.

	Target Group	Disability weight	Average age at onset	Duration in years (days)
Vitamin A				
Measles	Children < 5 years	0.051	/	.03(10 days)
Night blindness	Children < 5 years	0.031	/	1
Corneal Scarring	Children < 5 years	0.184	1	71.5
Bitot’s Spot	Pregnant Women	0.031	/	1
Bitot’s Spot	Women 15–49 years	0.031	/	1
Vitamin-D				
Rickets	Children < 5 years	0.023	/	.25(90 days)
Osteomalacia	Pregnant women	0.023	/	.25(90 days)
Osteomalacia	Women 15–49 years	0.023	/	.25(90 days)
			Age At death	Life expectancy
Mortality	Children < 5 years		1	71.5

A primary concern related to vitamin A deficiency is its link to child mortality. Various studies and multiple independent meta-analyses indicate a consistent association between vitamin A supplementation programmes, especially in undernourished settings or vulnerable population groups, and a statistically significant reduction in preschool child mortality, with estimates ranging from 23% to 34% [1,30–32]. Another meta-analysis, of randomized control trials in children aged six months to five years living in the community, yielded a weighted average mortality reduction of 12%. While a study in India estimated the mortality reduction to be around 4% [33,34]. Adopting a cautious approach, we used the values of 4%, 12% and 23% in our analysis.

Essential parameters for the Disability-Adjusted Life Years (DALY) formula, including disability weights and remaining life expectancies, were derived from the Global Burden of Disease (GBD) study and India life tables [18, 19, 35]. In line with standard practice in health impact analyses, a discount rate of 3% was applied to account for the present value of future health outcomes [19]. For the costing, we considered the following scenarios: (1) High-dose/massive dose vitamin A supplementation; (2) industrial fortification of oil with Vitamin A and D.

Additionally, we evaluated the cost savings from Vitamin D fortification for the elderly (60 years and above) by estimating the reduction in treatment expenses related to falls. Based on peer reviewed articles, the fall prevalence is assumed to be 31% among elderly and a risk reduction of 15% is expected in adequate Vitamin D doses are provided [36,37]. Cases of rickets and osteomalacia was assumed to be 10% among those suffering from Vitamin D deficiency. The cost of treatment for fall was assumed to be 1,00,000 rupees [38]. This figure is based on a study that estimated the cost of treating falls among elderly individuals aged 60 and above in rural areas of Tamil Nadu [38].

### High dose Vitamin A supplementation

We considered a massive dose Vitamin A supplementation (VAS) program providing semi-annual doses of 200,000 IU to children under 5 years [10]. The current vitamin A supplementation program in India concentrates on children up to 5 years. The Costs were calculated by multiplying the number of children up to 5 years and pregnant women by per capita costs. Census projection data was used to estimate the population figures [39]. The proportion of pregnant women was estimated using NFHS −5 data. In case of India, the National Prophylaxis Programme for Prevention of Blindness due to Vitamin A Deficiency in India was initiated in 1970 [10]. The cost for 100 ml of Vitamin A bottle and transportation from ware house to PHC was obtained from NHM [40]. As recommended by Government of India, we assume that each child under 5 years will require 17 ml (9 doses of Vitamin A, the first dose at 9 month is 1 ml) and each pregnant woman will require 3 ml (25000 IU week for 12 weeks) [10,41].

### Industrial fortification of oil and milk with Vitamin A and Vitamin D

The cost of fortification for oil and milk was (₹ 0.1) and (₹.02) respectively for 2016 [42,43]. Assuming a 4% inflation in milk prices and 5% inflation in oil prices, the revised cost of fortification was assumed to be USD.0018 (₹ 0.15) and USD 0.00036 (₹.03) respectively for 2024. Inflation rate is based on CPI index for milk and milk products and mustard oil. The value of CPI index was obtained from EPWRF database. The exchange rate was assumed to be ₹ 82.84 per $. The recommended amount of oil consumption per day by adults (women and elderly) and children is 30 g per day and 20 g per day respectively [44–46]. While the amount of milk consumed per day is recommended to be 500 ml per day for the target groups [47,48]. However, we used the estimates based on the National Sample Survey (2009−11). As per this survey, the monthly edible oil consumed per capita is 636 g in rural areas and 818 g in urban areas; monthly milk consumed per capita is 4.1 and 5.4 litre in rural and urban areas. The overall estimates were computed using a weightage of seventy percent for rural areas and thirty percent for urban areas. The cost of intervention was calculated by multiplying the cost of fortification of per kg of oil (per liter milk) with total consumption of oil (milk) for children and adults.

### Cost effectiveness

The cost effectiveness for the two scenarios was calculated by dividing the total cost by the DALYs [24].

## 3. Results

### Parameter values

The parameters which were used for the calculation of Disability-Adjusted Life Years (DALYs) are as follows (Table 1). Measles in children under 5 years has a disability weight of 0.051, and a duration of 0.03 years (10 days). Night blindness in children and Bitot’s spot in pregnant women has a disability weight of 0.031, and a duration of 1 year. Corneal scarring in children under 5 years has a disability weight of 0.184, an average age at onset of 1, and a duration of 71.5 years. For fatal conditions leading to mortality in children under 5 years, the average age is assumed to be 1 year, and the average life expectancy is 71.5. Rickets in children under 5 and osteomalacia among women have a disability weight of 0.023, and a duration of 0.25 years.

### Potential impact YLDs and YLLs averted

The potential rate of measles among under five is 193.5 per million, translating to 2,128 new cases and resulting in the averted Years Lived with Disability (YLD) of 3 (Table 2). Night blindness, with an incidence rate of 0.10%, corresponds to 109,965 new cases and leads to the substantial averted YLD of 3,358 (Table 2). Corneal scarring, with an incidence rate of 0.0022%, indicates 2,396 new cases and 12974 YLDs averted (Table 2). For pregnant women, Bitot’s spot has an incidence rate of 0.10%, resulting in an estimated 26,920 new cases and averted YLD of 822 (Table 2).

**Table 2:** Potential impact, YLDs averted, India.

	Total pop. Under five (in 1000s)	Incidence per MN	Incidence rate	Incidence rate *Pop.	YLD Averted
	${\text{T}}_{\text{j}}$		${\text{I}}_{\text{ij}}$	${{\text{T}}_{\text{j}}\text{I}}_{\text{ij}}$	
**Vitamin A**					
Children under five					
Measles	109965	193.5	19.4	2128	3
Night blindness	109965	0.100%	0.1000%	109965	3358
Corneal Scarring	109965	0.022%	0.0022%	2396	12974
Bitot’s Spot					
Pregnant Women	26920	0.100%	0.1000%	26920	822
Women (15–49 years)	372517	0.100%	0.1000%	372517	11377
	Live births (in 1000s)	Child mortality	Mortality rate	Mortality rate *Population	YLD averted
Mortality	${\text{T}}_{\text{j}}$		${\text{M}}_{\text{j}}$	${{\text{T}}_{\text{j}}\text{M}}_{\text{j}}$	
23%	23959	6.9	1.587	38023	1119044
4%	23959	6.9	0.276	6613	194616
12%	23959	6.9	0.828	19838	583849
**Vitamin D**	Population in 1000s	% deficient	% attributable to deficiency	Burden	
Children under five					
Rickets	109965	14	10%	1517515	8606
Osteomalacia					
Pregnant Women	26920	40	10%	1076809	6107
Women (15–49 years)	372517	40	10%	14900700	84505
	Population in 1000s	Falls prevalence (%)	Risk reduction (%)	Falls averted	
Elderly (60+)	153609	31%	15%	7142799	NA

The potential impact of reducing child mortality rates associated with improvement in Vitamin A levels by assuming following scenarios: 23%, 4%, and 12% reductions. Each region’s child mortality rate, calculated per 1000 live births, is multiplied by the corresponding population, yielding estimates of child deaths in each scenario. And the reduction in mortality owning to Vitamin A supplementation was applied to calculate the total number of deaths which could be averted (${{\text{T}}_{\text{j}}\text{M}}_{\text{j}}$). Assuming a 23%, 4% and 12% reduction in mortality, the number of deaths which could be averted in India are as follows: 38023, 6613 and 19838 respectively. The potential impact of a 23% reduction in mortality rates could avert 1,119,044 YLLs, while a 4% reduction could avert 194,616 YLLs, and a 12% reduction could avert 583,849 YLLs.

Regarding Vitamin D deficiency, 14% of children under five and 40% of women were Vitamin D deficient. It was assumed that only 1% of those suffering from Vitamin D deficiency will suffer from rickets and osteomalacia. Which translates into 1,517,515 cases of rickets and 8606 YLDs in children under five, 1,076,809 cases of osteomalacia and 6107 YLDs in pregnant women, and 14,900,700 cases of osteomalacia and 84,505 YLDs in women aged 15–49 years. If Vitamin D supplementation were provided to the elderly population, it could potentially prevent approximately 7,142,799 falls among the elderly.

### Supplementation cost

For children aged 1–5 years, the recommended dose is 200,000 IU, administered in 2 ml per dose, with a total of 9 doses recommended, resulting in 18 ml. The cost of a 100 ml Vitamin A bottle, inclusive of transportation to Primary Health Centers, is 110 rupees, converted to $1.32 at an exchange rate of 0.012$/rupee (Table 3). The cost of providing 9 doses (18 ml) per child is calculated at $0.24 or 19.9 rupees.

**Table 3:** Cost associated with supplementation.

Items	Cost and quantity
Recommended dose in IU for children under 5 years	2,00,000
In Millilitre (first dose is 1 ml)	2 ml
Total 9 doses recommended for under 5 years (in ml)	17
Cost 100 ml Vitamin A bottle (include transportation cost from warehouse to PHC) in ₹	110
Exchange rate (.012$/rupee)	82.84/$
Cost 100 ml Vitamin A bottle (include transportation cost from warehouse to the PHC) in $	1.32
Cost of providing 9 doses (18 ml) per child in $	0.24
Cost of providing 9 doses (18 ml) per child in ₹	19.9
Total requirement for pregnant women in IU (25000 IU week for 12 weeks)	300000
In Millilitre	3
Cost of providing 3 ml per PW in $	0.0396
Cost of providing 3 ml per PW in ₹	3.28
Total cost for 109965 thousand children under 5 in $	24.6 MN
Total cost for 26920 thousand PW in $	1.06 MN
Total cost for all beneficiaries in $	25.7 MN

Pregnant women, requiring 300,000 IU over 12 weeks, are administered 3 ml per week. The cost of providing 3 ml per pregnant woman is $0.0396 or 3.28 rupees. The total cost for providing vitamin A supplementation to 109,965 thousand children under 5 is approximately 24.6MN USD, while the total cost for 26,920 thousand pregnant women is approximately 1.06 MN USD. The total cost for providing supplementation to both children and pregnant women is around 25.7 MN.

### Fortification cost

Based on existing literature, the cost to fortify 1 kg of oil is.0018 USD (15 paise), while for 1 liter of milk is.00036 USD (3 paise) (Table 4). Monthly oil consumption is assumed to be 690 grams of oil and 4.5 liters per capita for milk. The annual consumption of oil in kilograms is 8.3 kg, and for milk is 53.7 liters, respectively. The cost to fortify is 7.6 million USD for oil and 9.8 million USD for milk fortification for children and women (Table 4).

**Table 4:** Cost associated with fortification.

	Oil fortification			Milk fortification		
	Children	Pregnant women	Women 15–49 years	Children	Pregnant women	Women 15–49 years
Cost to fortify 1 kg of Oil/1 litre of milk in ₹	0.15	0.15	0.15	0.03	0.03	0.03
Cost to fortify 1 kg of Oil/1 litre of milk in $	0.0018	0.0018	0.0018	0.00036	0.00036	0.00036
Monthly consumption oil in gm and milk in litres	690.6	690.6	690.6	4.5	4.5	4.5
Annual consumption of oil in kg and milk in litres for children/ for 12 weeks for pregnant women	8.3	8.3	8.3	53.7	53.7	53.7
Beneficiaries (in 1000)	109965	26920	372517	109965	26920	372517
Cost to fortify in MN (in USD)	2	0.4	5.6	2	0.5	7.2
Cost to fortify in rupees (in crores)	14	3.3	46	18	4.3	59.7

### Cost and cost-effectiveness

Vitamin A supplementation will cost 26 million USD, while fortification interventions for oil and milk will cost 9.5 million USD and 12.3 million USD (Table 5).

**Table 5:** Cost and cost-effectiveness of supplementation and fortification programmes.

	Children	Pregnant Women	Women (15–49 years)	Elderly	Total
Supplementation	25	1.07			
Oil Fortification	2	0.40	5.6	2.3	9.5
Milk Fortification	2	0.52	7.2	3	12.3
Total DALYs averted					
YLDs	24941	6929	95882		120824
YLLs (at 4% reduction)	194616				194616
DALYs	219557	6929	95882		315440
Falls averted				7142799	7142799
Monetary Value of DALYs in ₹ MN	25413	802	11098	714280	750791
Monetary Value of DALYs in $ MN	307	10	134	8622	9063
Cost benefit ratio Fortification	81	11	11	1639	416
Cost benefit ratio Supplementation	12.43	9.1			
Cost per DALY averted Fortification	17	133	133		
Cost per DALY averted Supplementation	112	154			

In terms of health impact, the total YLDs averted will be 24,941 YLDs for children, 6929 YLDs for pregnant women, and 95,882 YLDs for women aged 15–49 years. The YLLs averted due to a 4% reduction in mortality amount to 194,616 (Table 5).

The total DALYs averted are 219,557 for children, 6,929 for pregnant women, and 95,882 for women aged 15–49 years. Overall, a total of 315,440 DALYs would be averted. The economic value of the DALYs averted is $307 million for children, $10 million for pregnant women, $134 million for women aged 15–49 years, and $8,622 million for the elderly.

The cost benefit ratio for supplementation programme for children is 12.4, and for pregnant women and women is 9.1. The cost benefit ratio for fortification programme for children is 81, and for pregnant women and women (15–49 years) is 11. The overall cost benefit ratio of the programme is 416.

The cost of averting a DALY through the fortification programme is estimated at 17 dollars per DALY, and 133 dollars per DALY for children and pregnant women. The cost of averting a DALY through the supplementation programme is estimated to be 112 dollars per DALY and 154 dollars per DALY for children and pregnant women.

## 4. Discussion

In this study, an attempt has been made to evaluate the benefits and costs associated with two different interventions aimed at increasing the coverage of Vitamin A and Vitamin D supplementation among children under 5 years, women in the reproductive age group, and the elderly. Following are the five salient findings of this study. First, the study assessed the potential impact of reducing child mortality rates through improved Vitamin A levels, considering scenarios with 23%, 4%, and 12% reductions. Overall, these reductions could avert 1,119,044 Years of Life Lost (YLLs) at a 23% reduction, 194,616 YLLs at 4%, and 583,849 YLLs at 12%. Here, it is worth noting that the GBD estimates zero deaths related to Vitamin A deficiency for India. However, the GBD has changed the analytical methods, and the change in deaths compared to earlier years in the GBD might not reflect the true state [49]. Further, there have been several studies which attribute 4–35% of all the deaths under 5 years to Vitamin A deficiency [1,4,33,34]. Our results are in line with a similar study which estimated that Vitamin A supplementation and fortification could save 80–600 thousand lives over a 20-year horizon [17].

Second, the study examined the incidence rates of diseases such as measles, night blindness, corneal scarring, Bitot’s spot, rickets and osteomalacia, translating them into averted Years Lived with Disability (YLDs). Consumption of adequate Vitamin A could avert 2,128 new cases of measles, resulting in 3 YLDs. Night blindness, with an incidence rate of 0.10%, could avert 3,358 YLDs, while corneal scarring could avert 12974 YLDs. Bitot’s spot among pregnant women, with an incidence rate of 0.10%, could avert 446 YLDs. Consumption of adequate Vitamin D could avert 8606 YLDs of rickets and 84505 YLDs of osteomalacia. Here, it is worth noting that a previous study had estimated DALYs related to Vitamin A deficiency to be around 12–27 MN [17]. Our estimates are conservative as they factor in the increasing coverage of Vitamin A across different regions. It is anticipated that the current scenario has fewer deaths and fewer instances of poor functional outcomes compared to five years ago. Furthermore, we could not identify many cost-benefit studies assessing the impact of Vitamin D supplementation on health, indicating that there are limited studies on this subject matter.

Third, the cost of providing Vitamin A supplementation, recommended for children aged 1–5 years and pregnant women, was calculated. The total cost for supplying supplements to approximately 109,965 thousand children and 26,920 thousand pregnant women is around 26 million USD. The cost of fortifying oil and milk with Vitamin A and Vitamin D was evaluated based on existing literature. The overall cost to fortify oil and milk for children, Pregnant women and women aged 15–49 years is estimated to be 4, 0.9 and 12.8 MN USD, respectively. The cost is comparatively higher than that provided by NHM for states since we are assuming that supplementation will be provided to all the children and pregnant women irrespective of deficiency [40].

Fourth, the overall cost-effectiveness of supplementation and fortification interventions is estimated at 129 per DALY and 287 per DALY respectively for children and pregnant women. In terms of return on investment, the benefits are less than the per capita GDP of India, making the intervention extremely cost effective. These estimates are in line with some of the global estimates. For instance, a study in Tanzania found that the cost effectiveness ratio could be between 280 and 4000 USD per DALY averted [50]. The CEA ratio for China for fortified rice was estimated to be 5–18 USD per DALY averted [51]. For India, it was estimated to be 50 USD per DALY averted [17]. However, the estimates are likely to vary across countries depending on the extent of deficiency and cost of fortification. Further, it is worth noting that the consumption of oil and milk will have to be supplemented with consumption of other natural products to achieve the intake suggested by the Government of India with respect to Vitamin A. Consumption of oil and milk alone might not be sufficient.

Fifth, based on the assumption that Vitamin D supplementation is expected to reduce the risk of falls by 15%, a total of 7,142,799 falls can be averted among the elderly, which holds significant economic value. Here, it is worth noting that multiple reviews and meta-analysis show that Vitamin D helps improve muscle strength and balance, thereby reducing the likelihood of falls among elderly [52–54]. However, in case of severe deficiency, Vitamin D might have to be combined with calcium supplements.

This study is subject to several limitations. Firstly, the lack of recent data on the prevalence of various functional outcomes in India necessitated assumptions, particularly regarding the prevalence of night blindness, corneal scarring among children, and Bitot’s spot among pregnant women, all assumed to be 0.10. Similarly, the prevalence of rickets and osteomalacia is also not known. It is worth noting that a previous study based on the National Nutrition Monitoring Bureau survey indicated potentially higher cases of Bitot’s spot, especially in states like Uttar Pradesh and Bihar, but no recent estimates are available [17].

Secondly, the cost of fortification was derived from existing literature, as obtaining information directly from the industry proved challenging due to the fragmented nature of the oil industry, with numerous small-scale producers. It is not possible to provide a precise estimate. However, the government has suggested that the cost could be very low [42,43].

Thirdly, the study is limited by the lack of information on the coverage of Vitamin A and Vitamin D in the population. Consequently, the analysis focused on children under five and pregnant women. Although data on the number of doses administered is available in HMIS, information on coverage is not provided. To calculate coverage, we would need a reliable denominator such as the total number of eligible beneficiaries which is not provided by HMIS. Further, the benefits estimated could be an underestimate since only children and pregnant women were considered.

In conclusion, fortification could emerge as a potentially superior long-term solution, considering the widespread consumption of oil and milk, offering a broader reach to the population. Moreover, fortification presents the opportunity to incorporate other essential vitamins, potentially yielding substantial additional benefits beyond Vitamin A and Vitamin D alone. However, there is a need to conduct more evaluations using primary data to establish the benefits of fortification on health outcomes.

## Supporting information

S1 File Data used for estimation of costs and benefits. (ZIP)
